# Nonlinear Optical Microscopy and Plasmon Enhancement

**DOI:** 10.3390/nano12081273

**Published:** 2022-04-08

**Authors:** Yi Cao, Jing Li, Mengtao Sun, Haiyan Liu, Lixin Xia

**Affiliations:** 1 Liaoning Key Laboratory of Chemical Additive Synthesis and Separation, Yingkou Institute of Technology, Yingkou 115014, China; d202110417@xs.ustb.edu.cn; 2 School of Mathematics and Physics, University of Science and Technology Beijing, Beijing 100083, China; 3 Key Laboratory of Photochemical Conversion and Optoelectronic Materials, Technology Institution Physical and Chemistry, Chinese Academic Science, Beijing 100190, China; lijingsp@mail.ipc.ac.cn

**Keywords:** plasmon, nonlinear optics, microscopy systems

## Abstract

Improving nonlinear optics efficiency is currently one of the hotspots in modern optical research. Moreover, with the maturity of nonlinear optical microscope systems, more and more biology, materials, medicine, and other related disciplines have higher imaging resolution and detection accuracy requirements for nonlinear optical microscope systems. Surface plasmons of metal nanoparticle structures could confine strong localized electromagnetic fields in their vicinity to generate a new electromagnetic mode, which has been widely used in surface-enhanced Raman scattering, surface-enhanced fluorescence, and photocatalysis. In this review, we summarize the mechanism of nonlinear optical effects and surface plasmons and also review some recent work on plasmon-enhanced nonlinear optical effects. In addition, we present some latest applications of nonlinear optical microscopy system research.

## 1. Introduction

As one of the essential branches of modern physical photonics, the discovery of nonlinear optics benefits from the invention of lasers [1,2]. Nonlinear optics takes the physical phenomena and reality applications resulting from the interaction between glaring light and nonlinear materials as research goals, including the generation of harmonics, optical detection, frequency modulation, etc. [3]. In 1961, Fraken produced a light with a wavelength of 347 nm by irradiating a crystal with a ruby laser [4]. The wavelength of this new light is not the same as the wavelength of the incident light and is exactly half the wavelength of the incident light. This finding from the experiment verifies the second harmonic generation (SHG) for the first time. Since then, Schawlow et al., have also theoretically analyzed nonlinear optical phenomena such as quasi-phase matching and phase matching in the resonant cavity [5,6]. Effects such as stimulated light scattering, two-photon absorption, and optical Kerr effect are found in early nonlinear optics research [7,8,9]. The early discovery and exploration of nonlinear optical phenomena by researchers laid the foundation for the development of the field [10]. Based on the advantages of nonlinear optical effects, photonic devices such as nonlinear optical microscopes have matured [11,12]. However, due to the requirement of the high-intensity electric field, the inherent nonlinear optical response of the material is not obvious. Some nonlinear optical crystals usually use complementary ways to increase conversion efficiency, but they are challenging to use in fabricating integrated optoelectronic devices due to size constraints [13]. Therefore, how to improve the nonlinear response and conversion efficiency in the nanoscale is the current barrier to nonlinear optics research.

In 1921, Albert Einstein proved and revealed the existence of the photoelectric effect through experiments and won the Nobel Prize in Physics, which opened the research chapter of modern photoelectric interaction [14]. The unique reflective luster of the noble metal surface is closely related to the metal’s unique optical properties and the free charge shifting inside the metal [15]. When electromagnetic waves of a specific wavelength irradiate the metal nanostructures, the plasmon effect generated on the surface can bind the electromagnetic waves to the vicinity of the metal nanostructures [16]. Plasmonic nanostructures could control linear and nonlinear optical processes and enhance the interaction of light and material by confining electromagnetic waves in the subwavelength range, which has a wide range of application prospects in the fields of single-molecule detection [17], super-resolution detection [18], light capture and emission [19], and optical force manipulation [20]. Meanwhile, the plasmon effect’s modulation of nonlinear optical processes can also help improve the nonlinear optical effect under low-light conditions and reduce the size of nonlinear optical devices [21]. The discovery of these traits has a tremendous guiding effect on the research and development of integrated optoelectronic devices and has aroused the interest of researchers [22]. The generation of nonlinear optical effects relies on high-intensity external electromagnetic fields. Surface plasmons can trap light in nanostructures in free space and form huge local electromagnetic field enhancements [23]. Combining surface plasmons with nonlinear optical effects can realize nonlinear effects under low-light conditions and has excellent potential for developing nonlinear sensing and all-optical regulation.

This review starts from nonlinear optics and surface plasmons’ characteristics and introduces the research progress on the nonlinear optical effects of surface plasmon metal nanostructures. In addition, we also review the experimental results of the combined use of nonlinear optical microscopy systems and prospect the emerging application prospects of this field and the potential research directions for the existence of nonlinear optics in nanostructures.

## 2. Nonlinear Optics Effect Process of Surface Plasmon

**Figure 1:**
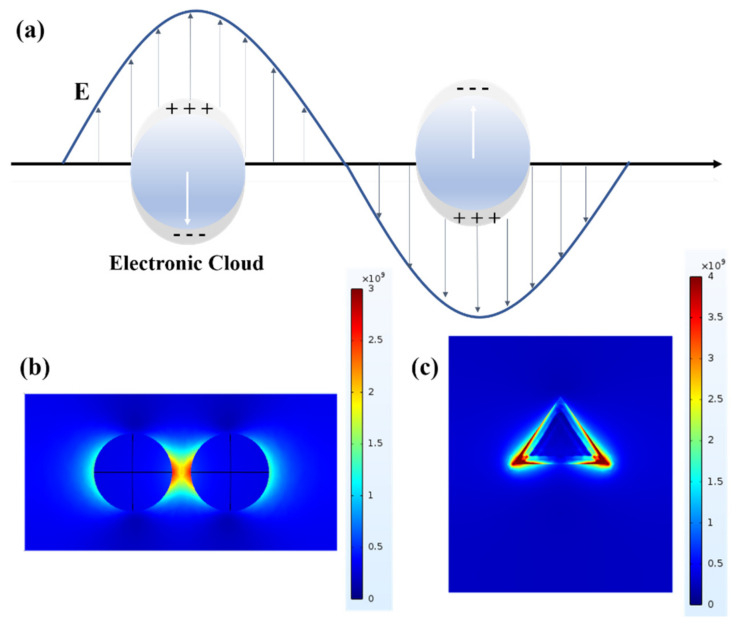
(**a**) Localized surface plasmon resonance on the surface of metal nanoparticles. (**b**) The hot spot with nanogap is when the two Au nanospheres with a diameter of 20 nm are separated by 3 nm. (**c**) The “tip-hot spot” effect at the tip of Au nanocones with regular tetrahedral structure.

Under the excitation of an external electric field, the free electrons located in the conduction band around the Fermi level in the interior of the metal nanoparticle will collectively oscillate on the particle surface. The electric field energy in the charge resonance state will be converted into the kinetic energy of the collective oscillation of free electrons on the particle surface and generate the surface plasmon (SP) effect, which includes the surface plasmon polariton (SPP) and localized surface plasmon (LSP) resonance [24]. Localized surface plasmon resonance refers to the collective oscillation of surface free electrons for metal nanoparticles under the excitation of an external field, resulting in boundary conditions due to the size and shape of the nanoparticles. Free electrons could resonate and enhance the local electric field strength around the metal nanoparticles under specific excitation conditions. The LSP peak intensity and position are affected by the morphology, size, material type, and dielectric constant of the metal nanoparticles, resulting in displacement or intensity change [25]. Assuming that there is an isotropic metal sphere with radius d inside the isotropic medium, and the radius d of the sphere is much smaller than the incident wavelength λ , it can be approximated that the electric field around the metal sphere is the same everywhere. In the other words, the model is a quasi-static approximation [26]. The extinction cross-section ($\sigma_{\mathrm{ext}}$) of the metal sphere is shown in Equation (1):

(1) $$\sigma_{\mathrm{ext}}=\sigma_{\mathrm{abs}}+\sigma_{\mathrm{sca}}=k\pi d^{3}12\varepsilon_{d}{}^{3/2}\left(\frac{\varepsilon_{m}^{\prime \prime }}{{\left(\varepsilon_{m}^{\prime }+2\varepsilon_{d}\right)}^{2}+\varepsilon_{m}^{\prime \prime }{}^{2}}\right)$$

where the wave vector k=2π/λ. $\sigma_{\mathrm{sca}}$ and $\sigma_{\mathrm{abs}}$ are the scattering cross-section and absorption cross-section of the metal sphere, respectively. $\varepsilon_{m}$ and $\varepsilon_{d}$, respectively, represent the complex permittivity of the metal sphere and the medium. When the $\sigma_{\mathrm{ext}}$ reach the most significant situation, most of the incident light is absorbed or scattered, which is the LSP resonance state. Complex permittivity of the metal sphere $\varepsilon_{m}=\varepsilon \left(\omega ,d\right)=\varepsilon_{m}^{\prime }\left(\omega ,d\right)+i\varepsilon_{m}^{\prime \prime }\left(\omega ,d\right)$, which means the complex permittivity of the metal sphere is closely related to the frequency of incident light and the size of metal particles. In addition, the imaginary and real parts of the complex permittivity correspond to the position and width of the LSP resonance peak, respectively. This indicates that the LSP resonance peak position and full half-high width could be regulated by changing the complex permittivity of the metal particles, which provides a way to study the controllability of LSP resonance. Researchers usually use chemical synthesis to prepare metal nanoparticles with controllable size and morphology and find that the curvature of metal nanoparticles is closely related to the enhancement of the local electric field [27]. In particular, nanotips, protrusions, and other “antenna”-like shapes have strong electromagnetic hot spots around them. In recent years, researchers have also found that the coupling effect of metallic nanogap can cause an extreme electric field enhancement in this region, which as shown in Figure 1. The local field strength variation law of electromagnetic coupling “hot spots” between metal nanoparticles by controlling the distance between metal nanoparticles [28].

Unlike LSP, surface plasmon polaritons refer to the resonant coupling of free electrons and incident photons on the surface of metal substrates with periodic structures such as metal nanodiscs and gratings to form an electromagnetic mode. In this case, evanescent waves are generated at the dielectric interface and propagate parallel to the interface [29]. SPP refers to a new hybrid electromagnetic mode generated by the resonant coupling of incident photons of an external field and free electrons on the surface of metal nanosubstrates, which propagates horizontally along with the interface between the surface of the metal substrate and the mediu [30]. The electric field amplitude decays exponentially along the vertical direction of the interface. In general, the field attenuation of air or glass above the metal is 1/2 of the incident light wavelength, and the metal’s attenuation depends on the surface depth of the metal. The real ($K_{r}$) and imaginary ($K_{i}$) parts of the SPP wave vector can be represented by Equations (2) and (3):

(2) $$K_{r}=\frac{\omega }{c}{\left(\frac{\varepsilon_{m}^{\prime }\varepsilon_{d}}{\varepsilon_{m}^{\prime }+\varepsilon_{d}}\right)}^{1/2}$$

(3) $$K_{i}=\frac{\omega }{c}\frac{\varepsilon_{m}^{\prime \prime }}{2\varepsilon_{m}^{\prime }{}^{2}}{\left(\frac{\varepsilon_{m}^{\prime }\varepsilon_{d}}{\varepsilon_{m}^{\prime }+\varepsilon_{d}}\right)}^{3/2}$$

where ω is the angular frequency of the incident light and *c* is the speed of light. $\varepsilon_{m}^{\prime }$ and $\varepsilon_{m}^{\prime \prime }$ are the real and imaginary parts of the metal dielectric constant and $\varepsilon_{d}$ is the dielectric constant of the medium. If the frequencies are the same, the plasmon wave vector on the metal surface is larger than the light wave vector ($K_{SPP}>\frac{\omega }{C}$). There will generate the SPP effect at the interface between metal and medium. Therefore, we must use the wave vector matching method to realize the resonance coupling excitation of surface plasmon and incident light.

**Figure 2:**
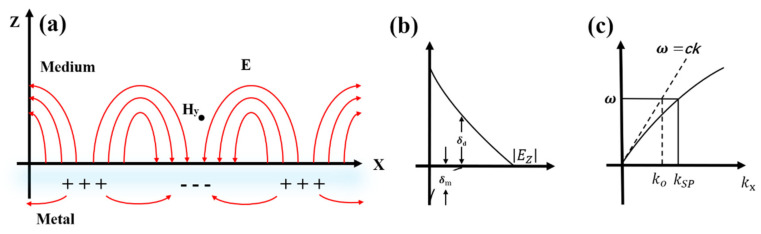
Electric field properties of SPP at the interface between dielectric and metal surface: (**a**) Coupling and electronic oscillation of SPP electromagnetic waves. (**b**) The electric field component of the SPP is bound on both sides of the interface and decays exponentially. (**c**) Dispersion plot of SPP.

The incident light wave must be excited on the metal surface to generate surface plasmon polaritons so that the effective control of the SPP mode can be accomplished. According to the SPP dispersion relationship in Figure 2c, it could be shown that at the same frequency, the photon wave vector is smaller than the surface plasmon polariton wave vector generated by exciting the metal surface [31]. Taken as a whole, only by increasing the wave vector of the incident light wave can the surface plasmon polaritons be excited on the metal surface.

The necessary condition for the generation of nonlinear optical effects is the interaction of high-intensity light with optic materials [32]. Nonlinearity in nonlinear optics means that the response of a substance to an external light field is in the form of a nonlinear equation, and is closely related to the intensity of the external light field [33]. For a material system, the relationship between the external light field intensity *E*(*t*) and the polarization intensity *P*(*t*) is shown by Equation (4):

(4) $$P\left(t\right)=P^{\left(1\right)}\left(t\right)+P^{\left(2\right)}\left(t\right)+P^{\left(3\right)}\left(t\right)+\cdots =\varepsilon \left[\chi^{\left(1\right)}E^{1}\left(t\right)+\chi^{\left(2\right)}E^{2}\left(t\right)+\chi^{\left(3\right)}E^{3}\left(t\right)+\cdots \right]\;$$

where ε is the dielectric constant in vacuum, and $\chi^{\left(n\right)}$ is the nonlinear polarizability of the *n* order. $P^{\left(1\right)}\left(t\right)$ is the linear part, where the resonance amplitude of the applied electric field is linearly related to the optical response of the materials. As the intensity of the applied light field increases, the nonlinear response of *P*(*t*) to the light field begins to appear gradually. Among them, the second-order nonlinear optical effect is closely related to $\chi^{\left(2\right)}$, which includes the second harmonic generation (SHG), sum-frequency generation (SFG), and difference frequency generation (DFG). The third-order nonlinear optical effects originating from polarizability $\chi^{\left(3\right)}$ mainly include triple harmonics generation (THG), four-wave mixing (FWM), coherent anti-Stokes Raman scattering (CASR), and so on. $\chi^{\left(n\right)}$ (5 *≤ n*) is the higher-order nonlinear optical process that usually produces higher-order harmonics generation (HHG). Among the common nonlinear optical processes, the nonlinear processes of $\chi^{\left(2\right)}$ and $\chi^{\left(3\right)}$ are relatively common, and the localized electromagnetic field enhancement caused by surface plasmon resonance could improve the conversion efficiency in nonlinear optics, so that has received extensive attention from researchers.

## 3. Surface Plasmon Resonances Enhanced the Nonlinear Optics Microscopy

### 3.1. Nonlinear Optical Enhancement in Surface Plasmons of Metal Nanoparticles

The essence of using surface plasmon resonance to enhance nonlinear optical effects is to match the frequency of the excitation light or frequency-doubling light with the surface plasmon resonance frequency to achieve the effect of excitation or emission enhancement [34]. Based on this condition, a variety of metal nanostructures were prepared by researchers, such as noble metal dimer structure [35,36], metal nanograting structure [37], metal nanocube structure [38], vertical Au nanorods (AuNRs) array structure, and so on [39,40]. Furthermore, for even-order nonlinear optical effects (e.g., SHG), the symmetrical structure of the material is essential [41]. Mi et al. [42] found that multi-surface plasmon resonance (MSPR) induced by Au@Ag NRs structure with frequency doubling effect could enhance CARS and two-photon excited fluorescence (TPEF) in nonlinear optical microscopy. By controlling the ratio of Hexadecyltrimethyl-ammonium bromide (CTAB), HAuCl_4,_ and AgNO_3_, they prepared Au@Ag NRs with different aspect ratios by wet chemical reduction method under a specific temperature and humidity environment. They successfully realized the frequency-doubling relationship of the UV-vis absorption peak positions of Au@Ag NRs. The fundamental and doubling peaks of Au@Ag NRs are at 800 nm and 400 nm, respectively. Meanwhile, they also demonstrated the enhancement effect of MSPR on nonlinear optical processes by TPEF and two-photon CARS characterization images of the two-dimensional (2D) material g-C_3_N_4_.

**Figure 3:**
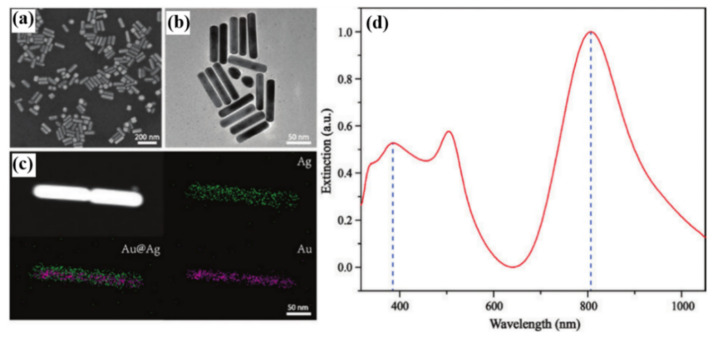
(**a**,**b**) Characterization image of Au@Ag NRs, respectively, by the scanning electron microscope (SEM) and transmission electron microscope (TEM). (**c**) Characterization results of different elemental compositions of Au@Ag NRs. (**d**) UV-Vis absorption spectra of experimentally synthesized Au@Ag NRs [42]. Republished with permission from Ref. [42]. Copyright 2022 Walter de Gruyter.

**Figure 4:**
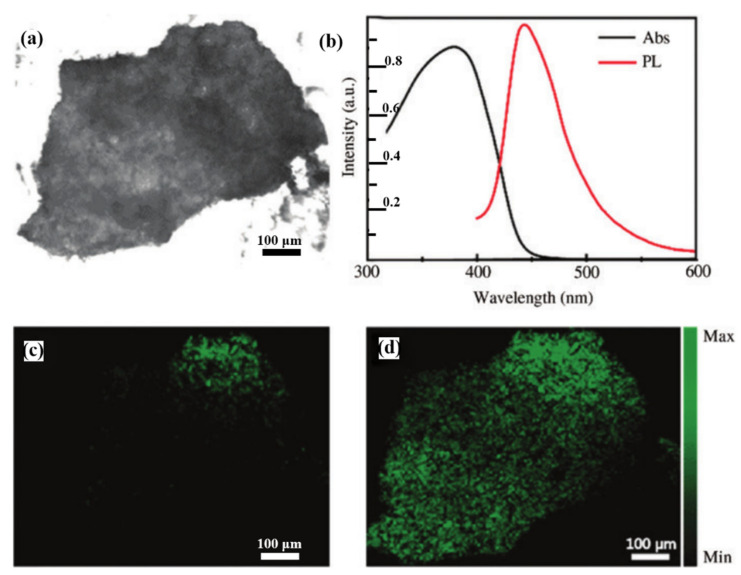
(**a**) Optical brightfield image of g-C_3_N_4_ without Au@Ag NRs.(**b**) Absorption and fluorescence (PL) spectra of g-C_3_N_4_. (**c**,**d**) are the TPEF images of g-C_3_N_4_ without and with Au@Ag NRs on the surface, respectively [42]. Republished with permission from Ref. [42]. Copyright 2022 Walter de Gruyter.

As shown in Figure 3a–c, they successfully coated a 10 nm-thick Ag shell on the surface of AuNR to form Au@Ag NRs. The length of the inner AuNR is 100 nm, and the diameter of the AuNR is 12 nm. Different from AuNRs, the emerging core-shell NR structure constructed by Au@Ag NRs will blueshift the resonance peak of AuNRs from 910 nm and 520 nm to 800 nm and 500 nm, and generate a new resonance peak at 400 nm, which is shown in Figure 3d. As a typical nonlinear optical phenomenon, TPEF approximates two high-intensity lower-frequency photons as one higher-frequency photon (frequency doubling relationship) to excite the fluorescence signal of the materials. The absorption peak of g-C_3_N_4_ in the 2D material is at ~400 nm, and the fluorescence emission peak is at ~450 nm, as shown in Figure 4b. Figure 4a is an optical microscopy image of g-C_3_N_4_ monolayer. Figure 4c,d are two-photon excitation (800 nm) fluorescence microscopy images of g-C_3_N_4_ and Au@Ag NRs/g-C_3_N_4_. Comparing the microscopic imaging results, it could be found that in Figure 4c, g-C_3_N_4_ only produces fluorescence under local focus, and the intensity is weak. In Figure 4d, there are Au@Ag NRs particles on the surface of g-C_3_N_4_. Based on the enhanced local electric field generated by the surface plasmon of Au@Ag NRs, the fluorescence intensity of g-C_3_N_4_ was significantly enhanced. The frequency-doubling absorption peak of Au@Ag NRs just matches that of g-C_3_N_4_, and the two photons could be absorbed in this band that realizes the fluorescence enhancement of g-C_3_N_4_. In addition, they also investigated the enhancement of CARS characterization of g-C_3_N_4_ by Au@Ag NRs, which is another nonlinear optical effect. The CARS and TPEF nonlinear optical signals of g-C_3_N_4_ can be distinguished after plasmon enhancement of Au@Ag NRs, because the frequency doubling of Au@Ag NRs at 800 and 400 nm enhances the non-linear optical signals. The simulations in COMSOL software strongly support the experimental results. At an incident angle of 30°, the enhancement factors (EF) of Au@Ag NRs surface plasmons for CARS and TPEF can reach $1.6\times 10^{4}$ and $6\times 10^{16}$, respectively, which is huge for the nonlinear optical signal enhancement of g-C_3_N_4_ alone. This research not only enables the enhancement of nonlinear optic signals by frequency doubling of metal nanorods but also demonstrates the great potential of MSPR for accurate characterization and improved imaging resolution in the future.

**Figure 5:**
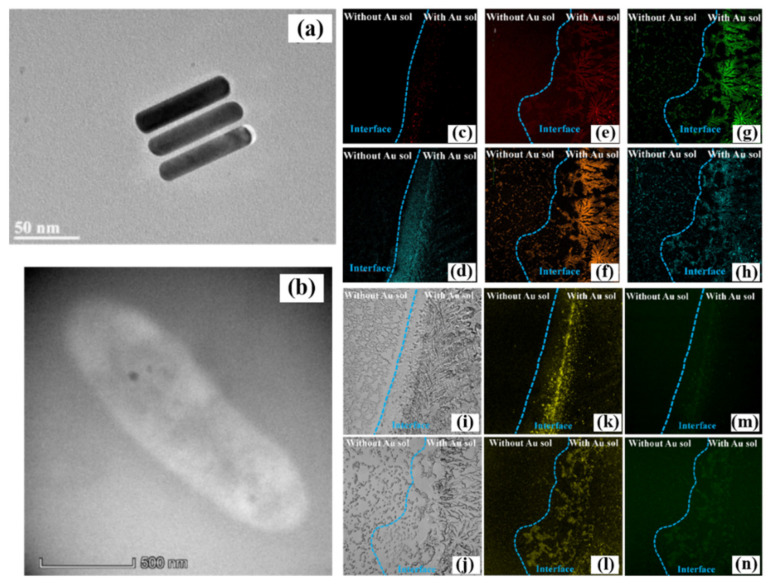
(**a**,**b**) are the TEM images of the self-assembled Au@Ag NRs and *E. coli*, respectively. (**c**,**d**) are the results of CARS imaging of *E. coli* at 1094 cm^−1^ and 2933 cm^−1^, respectively. (**e**–**h**) are the CARS imaging results of *S. aureus* at 1094 cm^−1^ (**e**), 1340 cm^−1^ (**f**), 1589 cm^−1^ (**g**) and 2933 cm^−1^ (**h**), respectively. (**i**–**n**) are the TPEF imaging signals of *E. coli* and *S. aureus* under brightfield microscopy (**i**,**j**), 570–630 nm (**k**,**l**), and 495–540 nm (**m**,**n**) channel regions, respectively. In the nonlinear microscopic imaging image, the right and left sides of the blue dotted line are the regions with and without Au@Ag NRs, respectively [43]. Republished with permission from Ref. [43]. Copyright 2022 Elsevier.

Based on the frequency-doubling relationship of Au@Ag NRs, Cui et al. successfully realized the nonlinear microscopic characterization of CARS and TPEF of plasmon-enhanced non-fluorescent microorganisms according to previous research, which is shown in Figure 5 [43]. They found that Au@Ag NRs with frequency-doubled surface plasmon resonance (SPR) peaks (400 nm/800 nm) could effectively induce the linear fluorescence signal of *E. coli* and also enhance the nonlinear spectral signal of its TPEF. Besides, the CARS nonlinear spectral signals of *E. coli* and *S. aureus* can also be effectively enhanced by the SPR effect of Au@Ag NRs. Figure 5a,b are the TEM characterization results of the Au@Ag NRs parallel array after the self-assembly experiment and the *E. coli* incubated in Au@Ag NRs solution, respectively. Between each self-assembly, Au@Ag NRs parallel array nanogap would generate the hotspot, which would enhance the local intensity of the electromagnetic field around the nanorod. They first performed confocal imaging of two standard CARS peaks of *E. coli* at 1094 cm^−1^ and 2993 cm^−1^ with light at 532 nm, as shown in Figure 5c,d. The left side of the blue dotted line is the colony area without Au@Ag NRs, and the right side is the colony area with Au@Ag NRs.

The imaging results clearly show that the nonlinear signal of *E. coli* CARS enhanced by the metal surface plasmon is more obvious, and the imaging resolution of the colony is greatly improved. They then also performed confocal imaging of the characteristic CARS signal of *S. aureus*. Similar results are shown in Figure 5e–h, the CARS imaging intensity on one side (right side of the blue dotted line) coated with Au@Ag NRs is significantly higher than that on the other side. This is because the SPR intensity of Au@Ag NRs is strongest at 800 nm, which can well enhance the different light in CARS. Meanwhile, it also proves that the surface plasmon of Au@Ag NRs indeed enhances the CARS signal. In order to better prove that plasmons can enhance nonlinear optical efficiency, they also verified the TPEF process of *E. coli* and *S. aureus* by using the plasmon frequency doubling effect of Au@Ag NRs. Analyzing the microbial environment and activity using CARS technology is cumbersome, and the fluorescent metabolite signals of microorganisms are more commonly used in research to confirm the life state of microorganisms. Figure 5i,j, respectively, are bright-field microscopic images of *E. coli* and *S. aureus*, from which the distribution of colonies and the effect of Au@Ag NRs on colony growth could be observed. Figure 5k–n are the TPEF confocal images of *E. Coli* and *S. Aureus* in the 575 nm to 630 nm and 495 nm to 540 nm channels under two-photon excitation at 800 nm, respectively. The blue dotted line is also used as the coverage with or without Au@Ag NRs. The imaging results could clearly demonstrate the signal amplification in the nonlinear optical process of two-photon fluorescence imaging by plasmon-enhanced. The research results provide a promising prospect for applying frequency-doubling metal nanoparticles in plasmon-enhanced nonlinear optical processes and microscopic imaging.

**Figure 6:**
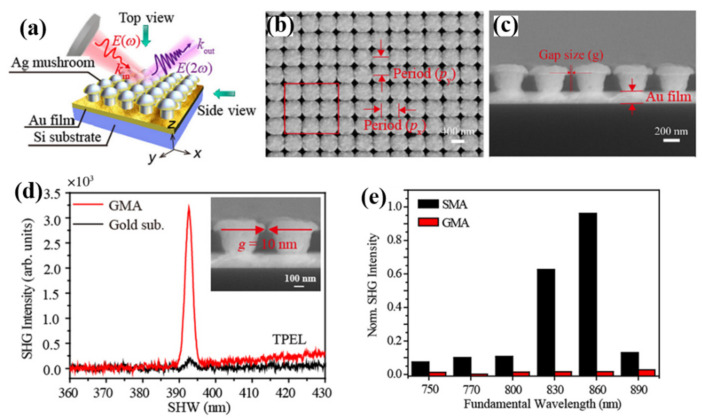
(**a**) Schematic diagram of the fabrication of metal mushroom arrays. (**b**) The top view of SEM characterization of SMA. (**c**) A single Ag mushroom has a height of 220 nm and a diameter of 300 nm, which is the side view. (**d**) Signal comparison of GMA and SHG on the surface of Au film substrate. TPEL is expressed as two-photon excitation should. Inset is the SEM characterization of GMA. (**e**) Fundamental wavelength-dependent comparison of PESHG performance of SMA and GMA [44]. Republished with permission from Ref. [44]. Copyright 2022 American Physical Society.

Shen et al. [44] designed a reusable plasmon-enhanced SHG (PESHG) substrate suitable for near-ultraviolet (NUV) wavelengths synthesizing Ag mushroom arrays. As shown in Figure 6a, they fabricated Ag mushroom arrays on Si/Au film substrates using nanolithography imprinting technology and the electrochemical deposition process. Figure 6b,c are the SEM characterization results of this array, which intuitively illustrate the uniformity of the array, and this periodic structure is the key to the SPP effect. In addition, controlling the nanogap spacing between mushrooms also helped generate more electromagnetic hotspots. Metal mushroom arrays are characterized by reducing their centrosymmetry at the three-dimensional level and can significantly enhance the SHG signal. Compared with the SHG signal generated by the surface of Au film alone, the SHG signal released by the Au mushroom array (GMA) was enhanced by 13 times, as shown in Figure 6d. Benefiting from the superior metal activity of Ag compared to Au, they deposited an Ag film on the surface of the Au mushroom array (SMA). They found that the new Ag mushroom array improved the SHG signal significantly. Under different fundamental frequencies, SMA has improved SHG signal than GMA, as shown in Figure 6e, especially at the fundamental wavelength of 860 nm, it has a nearly 80-fold improvement. This finding has guiding significance for applying SHG in nonlinear optics, such as optical communication and surface detection.

**Figure 7:**
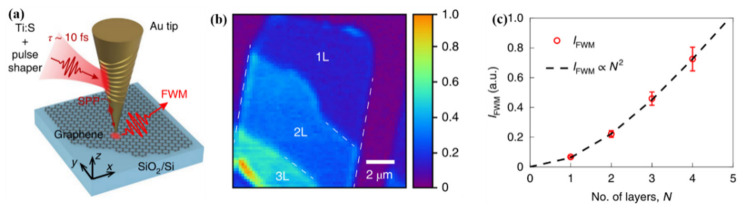
(**a**) Schematic illustration of the Au tip after grating etching for localized FWM excitation. (**b**) Near-field FWM imaging of monolayer and bi- and tri-layer graphene. The white dotted line is the edge of the graphene. (**c**) Fitted model of the dependence of thickness (*n*) on the intensity of integrated FWM (*I_FWM_*) [47]. Republished with permission from Ref. [47]. Copyright 2022 Springer Nature.

Apart from the surface plasmon effect produced by common metal nanoparticles (nanorods, nanoparticles…), metal tips as a typical metal structure, produce a huge localized electromagnetic field focus effect, which is widely used for scanning probe microscopy (SPM), tip-enhanced Raman scattering (TERS) and other research [45,46]. Jiang et al. [47] achieved coherent nonlinear imaging and graphene nanospectroscopy detection by means of metal tip excitation. They achieved the SPP effect by etching the periodic grating structure on the outer ring of the Au tip, as shown in Figure 7a, and a huge local electromagnetic field was generated at the Au tip with a radius of 10 nm, so they were able to detect the four-wave mixing (FWM) response of graphene. Figure 7b shows the imaging results of different layers *n* of graphene according to different response strengths.

As *n* increases, the strength (*I_FWM_*) of the FWM then exhibits a quadratic dependence ($I_{FWM}\propto N^{2}$), which is shown in Figure 7c. Based on this research, the number of graphene layers could be judged according to the value of *I_FWM_*. More essentially, this study demonstrates the feasibility of near-field nonlinear optics research in the nanometer range. Among them, the increase in nonlinear signals due to nano-focusing and near-field enhancement enables the further development of nonlinear nano-optics of two-dimensional materials and their nanostructures. This also makes it possible to finally develop nonlinear optical integrated devices with ultra-high sensitivity.

**Figure 8:**
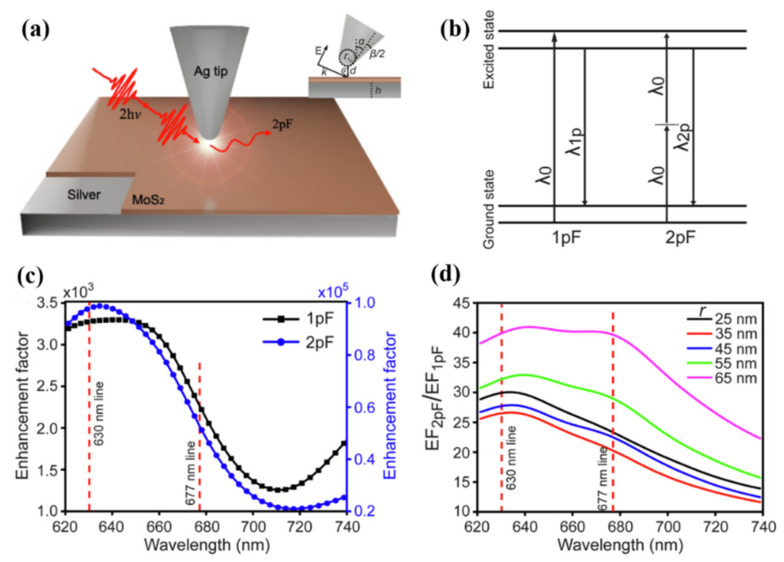
(**a**) Model of the 2 pF process on the Ag tip with the MoS_2_ surface. (**b**) Energy level diagrams of excitation and emission processes of 1 pF and 2 pF. (**c**) Comparison in enhancement factors of 1 pF and 2 pF on MoS_2_ surface. (**d**) The change in the Ag tip radius couples the SPR and enhances the exciton emission peak at 677 nm [48]. Republished with permission from Ref. [48]. Copyright 2022 Elsevier.

MoS_2_ as a transition metal dichalcogenide (TMDs), has similar physical structure and chemical properties to the typical two-dimensional material graphene and is currently a research hotspot in the field of semiconductors and two-dimensional materials. Wang et al. used a finite-difference time-domain (FDTD) method to investigate the properties of the MoS_2_ monolayer with the Ag tip to enhance two-photon-excited fluorescence (2 pF) [48]. Figure 8a shows the model structure of MoS_2_ tip-enhanced by 2 pF. The incident light in the model is irradiated to the MoS_2_ surface with a thickness of 1 nm on the Ag substrate by oblique incidence. The inset is a detailed schematic of the model. Among them, the incident angle is θ, the tip radius is r, the tip full cone angle is β, and the tip inclination angle and spacing are denoted by α and d, respectively. In the general fluorescence process, due to the low photon density of the incident light, a fluorescent molecule can only absorb one photon simultaneously and then emit a fluorescent photon through the radiation transition, which is called single-photon fluorescence.

For the two-photon fluorescence process, the intensity of the excitation light source is relatively high, and the photon density meets the requirement of simultaneous absorption of two photons by fluorescent molecules. In the normal two-photon fluorescence emission process, the photon density is insufficient to produce two-photon absorption, so a femtosecond pulsed laser is usually used, and its instantaneous power can reach the order of megawatts. The wavelength of two-photon fluorescence is shorter than that of the excitation light, but the photon energy density is higher, which is equivalent to the effect produced by half-excitation wavelength excitation, which is shown in Figure 8b, which is a schematic diagram of the energy level of 2 pF. Compared with the single-photon fluorescence emission, the high-order nonlinear optical process in the tip-enhanced 2 pF process leads to the greatly enhanced fluorescence emission. As shown in Figure 8c,d, the 2 pF overall enhancement factor of MoS_2_ under Ag tip enhancement is much higher than 1 pF, and a distinct fluorescence emission peak can be observed at the 630 nm exciton transition. To enable resonance matching of the SPR with the exciton transition process at 677 nm, a maximum enhancement of 2 pF at r = 65 nm was achieved by varying the radius of the Ag tip (25 nm to 65 nm) and was 41 for EF*_2 pF_*/EF*_1 pF_*. This shows that the strength of 1 pF can be increased by at least 40 times using the 2 pF process of the tip to MoS_2_. These works also show that the metal tip in the SPM can improve the conversion efficiency of nonlinear optical processes, achieve coupling, and enhance the strength of nonlinear optical signals under specific conditions.

### 3.2. The Application of Nonlinear Optical Microscopy System

Nonlinear optical microscopy, as a type of laser scanning microscopy, is to image the optical signals generated by the nonlinear interaction between incident light and the sample. There is a nonlinear relationship between the size of the imaged optical signal and the intensity of the incident light. Compared with traditional linear optical microscopes, nonlinear optical microscopes have the advantages of long-wavelength excitation, high spatial resolution, and ultrafast lasers as light sources that could reduce the average power of laser pulses. In recent years, nonlinear optical microscopy systems (such as CARS, SHG, and TPEF microscopy imaging techniques) have been widely used in biological sciences, medical research, and nanomaterials research and development [49]. Among them, the study of SHG signals on the surface of transition metal dichalcogenides (TMDCs) with a certain torsion angle and at the heterojunction based on the polarization-dependent nonlinear microscopies presents a possibility for the application of nonlinear optical microscopy in surface optical inspection [50,51]. In this section, we focus on reviewing the work of nonlinear optical systems in practical applications.

**Figure 9:**
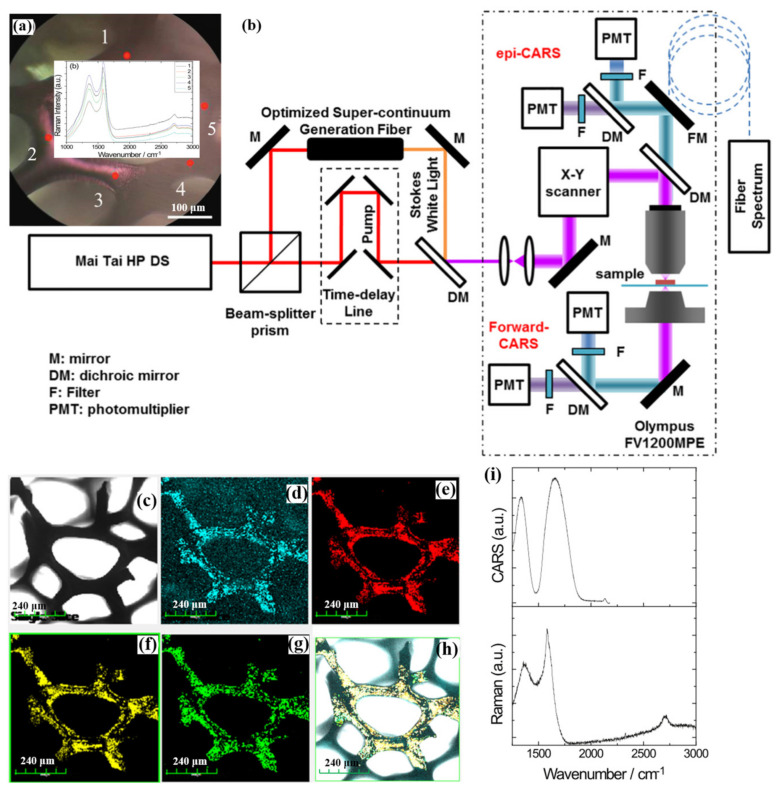
(**a**) Bright-field microscopic image of porous carbon after thin nickel structure. Insets are Raman spectral images at five different points. (**b**) Optical path diagram of the nonlinear optical microscope system. (**c**) Brightfield images of porous carbon materials. (**d**) SHG image of porous carbon materials. (**e**,**f**) are CARS images at 1587 cm^−1^ and 1360 cm^−1^, respectively. (**g**) is a TPEF image of the porous carbon material. (**h**) is the merged image of Figure 9a–e. (**i**) compares porous carbon material CARS with Raman spectroscopy [52]. Republished with permission from Ref. [52]. Copyright 2022 Elsevier.

Li et al. imaged porous carbon structures by building the nonlinear optical microscopy system [52]. The specific optical path of the nonlinear optical microscope system is shown in Figure 9b. Figure 9a shows an optical image of the porous carbon after the removal of the nickel structure, in which the synthesis of the porous carbon is completed on the surface of the nickel structure, and the nickel structure has been removed in the optical measurement. The inset in Figure 9a is the Raman signal measured at five different points in the optical image, which can demonstrate the reproducibility and homogeneity of the Raman spectrum of the porous carbon material. Figure 9c,d are the bright-field images and SHG imaging results of the porous carbon material, respectively. The structure of the porous carbon material can be clearly distinguished by comparison. Optical transitions and scattering can be performed in non-centrosymmetric media due to nonlinear optical effects. The stacking of multilayer porous carbon structures could induce SHG generation. Moreover, when the multilayer material is grown epitaxially, the perturbation of the Dirac cone causes the bandgap to open. This indicates that the centrosymmetry is broken, and thus the second-order nonlinear optical response of the porous carbon material can be enhanced. Figure 9e,f, respectively, are the CARS imaging results at 1587 cm^−1^ and 1360 cm^−1^. Figure 9g shows the imaging signal and image structure of the porous carbon material TPEF, which is clearly showing the porous carbon material structure. The transfer of photon momentum in TPEF to the electron system is also demonstrated. Figure 9h is the combined image of Figure 9a–e. The upper and lower figures in Figure 9i are the characterization comparison of CARS and Raman spectra of porous carbon materials, respectively. The nonlinear optical microscope system can reveal that the optical properties of porous carbon materials can be well revealed by the nonlinear optical characterization results of CARS, SHG, and TPEF by the nonlinear optical microscope system. The nonlinear optical microscopy system also provides a potential channel for studying other micro and nanoscale materials. Sun et al. studied the nonlinear optical response of single-layer graphene based on this nonlinear optical system [53]. They found that these nonlinear microscopes could not only clearly observe the morphology and structure of single-layer graphene but also effectively evaluate the quality of graphene. This research shows unique advantages in applying 2D material characterization and biological and medical research.

**Figure 10:**
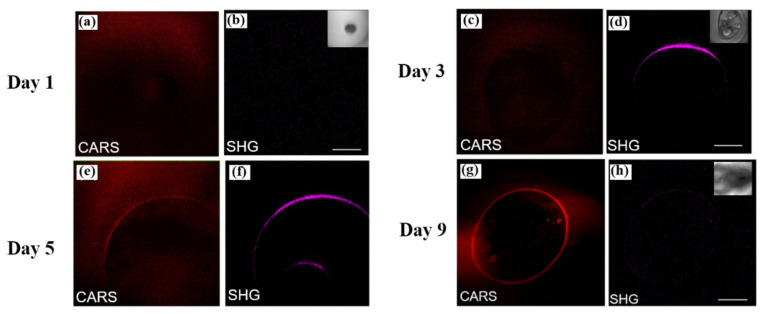
(**a**,**b**), (**c**,**d**), (**e**,**f**), (**g**,**h**) are the imaging results of CARS of snail bone elements and SHG of collagen on days 1, 3, 5, 9, respectively. Inset shows brightfield microscopy images of snail evolution on the corresponding day [55]. Republished with permission from Ref. [55]. Copyright 2022 John Wiley and Sons.

In the process of medical diagnosis and treatment, drug screening and avoidance of side effects are essential research directions. However, traditional screening processes require molecular labeling and detection of fluorescence data. This method of detection carries the risk of altered biological activity. Francesco et al. developed a label-free imaging system based on nonlinear optical microscopy [54]. The nonlinear imaging microscope system combines CARS and Bessel beams to ensure high-resolution spectral imaging and chemical composition analysis at the same time. Based on the nonlinear optical microscopy system, they studied the side effects of drug-induced lipid storage in liver tissue, and successfully extracted the spectral and spatial distribution of lipid and protein components, providing a new channel for detecting drug reaction side effects. Li et al. used nonlinear optical microscopy to observe in situ the symbiosis and competition between collagen and bone during biological evolution [55]. Using nonlinear optical microscopy, they successfully observed that sdsCO_3_^−2^ and PO_3_^−2^ induced collagen production and bone formation. As bones mature, collagen is gradually broken down from around the bones. Using CARS and SHG nonlinear optical microscopy techniques, the evolution and degradation of bone and collagen can be monitored during snail growth. Figure 10 shows the results of in situ characterization of snail bone and collagen from the first, three, five, and nine days after birth. As shown in Figure 10a,b, a large number of sdsCO_3_^−2^ and PO_3_^−2^ on day 1 are clustering together with the CARS imaging, while the SHG signal of collagen is not very obvious. The inset is the brightfield microscopic image of the snail corresponding to the day. In the CARS imaging results of sdsCO_3_^−2^ and PO_3_^−2^ on day 3, it can be found that the aggregated sdsCO_3_^−2^ and PO_3_^−2^ are forming bone contours. At the same time, collagen also grows along the outline of the bone with the aggregation of sdsCO_3_^−2^ and PO_3_^−2^. On day 5, the outline of the snail’s bones gradually became clear, and the texture began to become apparent. With the rapid development of bones currently, the content of collagen also reaches its peak. On day 9, the snail’s skeletal outlines were clearly visible in the CARS imaging, and the circular outlines were fully visible. However, due to the inverse feedback and biological competition mechanism, the SHG signal of collagen at this time degenerates and disappears due to bone maturation. The experimental results brilliantly reveal that the biological evolution of snail bones and collagen has been imaged over time. Based on the study of the synergistic and competitive relationship between collagen and bone growth, it is helpful to explore the repair mechanism of fractures or other bone diseases. The synergistic imaging of these nonlinear optical effects in vivo is helpful to observe the interaction between various substances in the organism.

**Figure 11:**
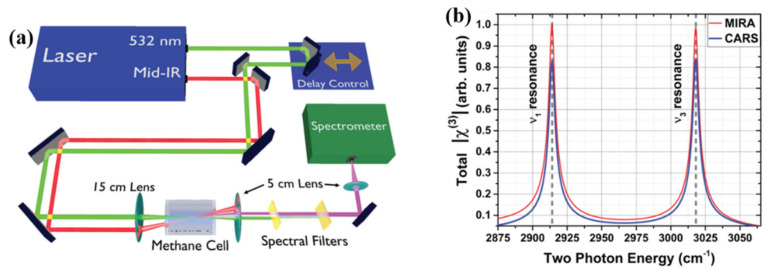
(**a**) The optical path of CARS nonlinear optical system based on MIRA. (**b**) $\chi^{\left(3\right)}$ as a detuning function of MIRA and CARS [56]. Republished with permission from Ref. [56]. Copyright 2022 American Physical Society.

In order to improve the imaging and detection level, Andrew et al. proposed a CARS nonlinear optical system based on Mid-IR assisted (MIRA) [56]. The nonlinear optical system can effectively obtain coherent Raman scattering signals in a low-noise background, which making the microscope system a potential option for highly sensitive spectral characterization and high-precision imaging. Figure 11a is a schematic diagram of the optical path of the entire nonlinear optical microscope system. They focused two optical signals (532 nm and IR) into methane gas, and then coupled the Raman signal generated after excitation into a spectrometer for analysis. Taking the amplitude of $\chi^{\left(3\right)}$ as the detuning function of IR-enhanced Raman and CARS can reflect the enhancement of Raman signal more realistically. The result after they added $\chi^{\left(3\right)}$ to the Raman signal is shown in Figure 11b. Compared with traditional CARS, MIRA-based CARS can more effectively couple the ground and excited states. With the advantages of this system, the MIRA-based CARS nonlinear optical microscope system provides a new solution for spectral detection and imaging with high sensitivity.

As the main energy substance of the human body, glucose is often used in solvent medicines or energy supplements and plays a significant role in the synthesis and catabolism of cells. The metabolism of glucose itself is closely related to the metabolic decomposition in various pathological reactions, and it is often used in the early detection technology of cancer. Long et al. proposed a two-color stimulated Raman scattering (SRS) imaging method to detect glucose uptake and metabolic activity in mouse brain tissue [57]. As a nonlinear process in optical inelastic scattering, SRS enables high-resolution imaging of probe substrates. By editing ^13^C, they constructed a ^13^C-labeled 3-*O*-propargyl-_D_-glucose (3-OPG-^13^C_3_) probe with a new Raman characteristic peak to ensure the visual monitoring of glucose uptake by the original 3-OPC probe in vivo. Moreover, the probe can achieve spectral resolution for other types of glucose (D7-glucose).

**Figure 12:**
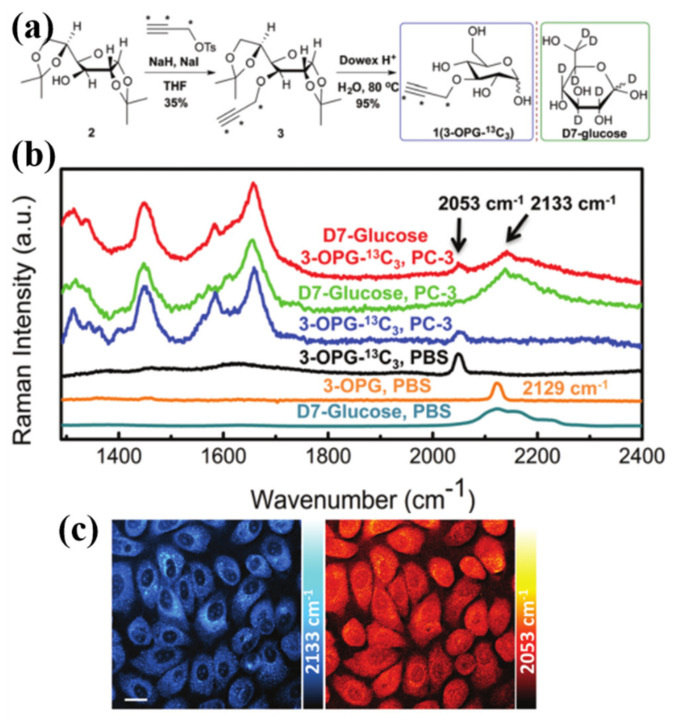
(**a**) The editing and synthesis process of 3-OPG-^13^C_3_ and the structure of D7-glucose. (**b**) Raman spectra of different types of glucose probes in PC-3 and PBS solutions. (**c**) Two-color SRS imaging of PC-3 cells using 3-OPG-^13^C_3_ and D7-glucose probes at 2133 cm^−1^ and 2053 cm^−1^, respectively [57]. Republished with permission from Ref. [57]. Copyright 2022 Royal Society of Chemistry.

Figure 12a is their flow chart of experiment. Inspired by the strategy of isotope editing in vibrational spectroscopy, they successfully synthesized 3-OPG-^13^C_3_ with total productivity is 33%, and the 3-OPG-^13^C_3_ probe does not have any crosstalk with the spectrum of D7-glucose. This new combinatorial probe enables the study of glucose uptake activity and synergy in adipogenesis by SRS imaging. Figure 12b shows the Raman spectra of different kinds of probes in prostate cancer cell-3 (PC-3) and PBS dispersion, respectively. Among them, the red chromatographic line is double-labeled with D7-glucose and 3-OPG-^13^C_3_ probes in PC-3 solution, and two different peaks are generated at 2053 cm^−1^ and 2133 cm^−1^, which represent the uptake and metabolism of glucose, respectively. The Raman characteristic peaks of 3-OPG-^13^C_3_ reflect the uptake of glucose in PC-3, while the characteristic peaks of D7-glucose reflect the metabolism of glucose in PC-3. Edited combinatorial probes are used for simultaneously studying glucose uptake and incorporation activity using SRS microscopy. This research provides a practical experimental idea for the diagnosis and treatment of cancer.

**Figure 13:**
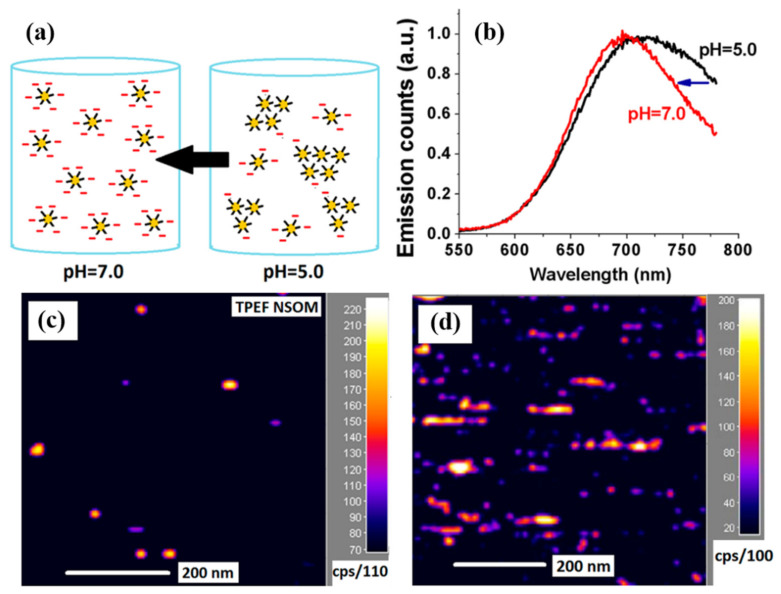
(**a**) Changes in pH lead to changes in the spacing of Au_25_ nanoclusters. (**b**) The increase in pH narrows the change in the full width at half the maximum of the emission peak of the molecule. (**c**,**d**) are the TPEF imaging results of 1.4 nm and 12 nm solutions coated on the glass substrate surface, respectively [58]. Republished with permission from Ref. [58]. Copyright 2022 American Chemical Society.

To achieve high-precision characterization to break through the diffraction limit is one of the research directions of micro and nano microscopic systems when studying substances below the optical diffraction limit. Neranga et al. achieved TPEF imaging of single and nanoclusters (NC) glutathione-protected Au_25_ (Au_25_SG_18_) by near-field scanning optical microscopy [58]. They adjusted the pH in the Au_25_SG_18_ solution to induce a change in the charge environment in the solution, which facilitated the separation of single Au_25_SG_18_ NCs, as shown in Figure 13a. This is due to the increased emission density caused by the aggregation of Au_25_ NCs at pH = 5.0. Broader emission spectrum compared to pH = 7.0 solution as shown in Figure 13b. To further verify the effect of solution concentration on the TPEF imaging of NSOM, they selected 1.4 nm and 12 nm solutions for TPEF imaging control, which is shown in Figure 13c,d. The nature of the different imaging results is related to the size of the spacing between the nanoclusters. The imaging results in Figure 13c demonstrate that TPEF for single NCs can be achieved by NSOM when the nanocluster spacing is less than 50 nm. Taking advantage of the unique optical properties of Au_25_, the nanoenvironment can be assessed by non-optical microscopy of TPEF for local ultrasensitive detection. Moreover, a single Au_25_SG_18_ NC can be used in a biosensing system and image detection of the local nanoenvironment in the organism, thereby realizing the early diagnosis of disease.

**Figure 14:**
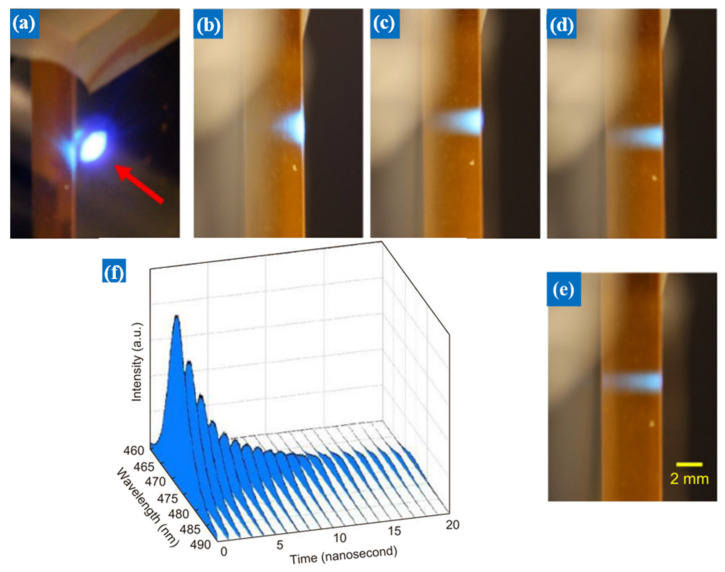
The two-photon-induced fluorescence of polycrystalline ZnSe at 775 nm is dependent on the incident laser intensity. (**a**) Blue fluorescence front view of polycrystalline ZnSe. (**b**–**e**) are the side views of the fluorescence radiation at incident light intensities of 25GW cm^−2^, 4GW cm^−2^, 1.5GW cm^−2,^ and 1GW cm^−2^, respectively. As the incident intensity decreases, the depth of fluorescence radiation in the sample becomes larger. (**f**) is the time-dependent decay curve of polycrystalline ZnSe with fluorescence intensity and wavelength [59]. Republished with permission from Ref. [59]. Copyright 2022Chinese Academy of Sciences.

ZnSe doped with a single crystal structure is a popular choice for the preparation of light-emitting diodes, and its optical properties exhibit nonlinear response characteristics with the change of incident light intensity. Irradiation of single-crystal ZnSe in the near-infrared band (~770 nm) with a femtosecond laser results in a two-photon absorption process followed by blue high-energy fluorescence photons (~475 nm). However, single-crystal ZnSe is not easy to obtain, and the preparation cost is higher than that of polycrystalline structure. Geoff’s research group studied the two-photon absorption process of polycrystalline ZnSe, and considered whether polycrystalline ZnSe could induce stimulated emission through two-photon absorption at 775 nm under ultra-high light intensity [59]. Due to the short fluorescence lifetime, a 0.5 mm thick ZnSe thin layer sample was mounted in an optical cavity with a length of 10 cm. As illustrated in Figure 14a, a femtosecond laser was used to irradiate a polycrystalline ZnSe material with high-intensity incident light with a wavelength of 775 nm, and the two-photon-induced fluorescence effect was successfully achieved. Figure 14b–e shows that the researchers found that the fluorescence radiation depth of polycrystalline ZnSe gradually increased under different incident light intensities. Under long pulses, as the peak intensity of the incident light intensity diminishes, the fluorescence intensity also gradually decreases and penetrates deeper into the material. Figure 14f shows the lifetime fluorescence intensity versus wavelength decay with time through the nonlinear optical microscopy system. It could be found that the fluorescence lifetime decays gradually with the increase in the pumping intensity of the incident light, which indicates that energy exchange is taking place between the ions. These findings demonstrate that polycrystalline ZnSe could reach the lasing threshold by two-photon pumping at 775 nm using a femtosecond laser under appropriate pumping intensity and crystal cooling conditions. This research has great commercialization potential in developing fluorescent display and lighting equipment.

## 4. Summary

The nonlinear optical effect will make the imaging focal spot smaller than the focal spot of the excitation light, thus breaking the classical diffraction limit, greatly improving the spatial resolution of confocal microscopy, and enabling super-resolution three-dimensional imaging. In particular, the surface plasmon of metal nanoparticles based on the frequency doubling effect can amplify nonlinear optical signals, improve the efficiency of nonlinear processes, and enhance the imaging resolution of nonlinear optical microscopes. This review analyses and summarizes the mechanism of plasmon enhancement of nonlinear optical signals by introducing and commenting on related research work. In addition, the applications and prospects of nonlinear optical microscopy systems in biology, medicine, and materials research are reviewed in this paper. These studies and applications attempted in practical scenarios will profoundly impact the update and development of nonlinear optical microscopy systems in the future.

## 5. Outlook

The electromagnetic field enhancement and hot electron transfer in surface plasmons can enhance nonlinear optical effects, especially the combination of some surface plasmon coupling structures and semiconductor materials can significantly improve the nonlinear conversion efficiency. Although a large number of research and theoretical explorations are currently striving to build a complete system for enhancing the conversion efficiency of nonlinear optics, there are still some physical mechanisms that are still being explored and debated. We believe that the system for nonlinear optical enhancement mechanisms will become more and more mature shortly, which is also crucial for the construction of nonlinear optoelectronic devices.

## Data Availability

The data presented in this study are available on request from the corresponding author.
